# Targeting ion channels: innovative approaches to combat cancer drug resistance

**DOI:** 10.7150/thno.103384

**Published:** 2025-01-01

**Authors:** Qian Shi, Zijing Yang, Huan Yang, Lihui Xu, Jing Xia, Jie Gu, Mengting Chen, Yan Wang, Xiaohong Zhao, Zehua Liao, Yiping Mou, Xidong Gu, Tian Xie, Xinbing Sui

**Affiliations:** 1 School of Pharmacy, Hangzhou Normal University, Hangzhou, Zhejiang, China.; 2 Department of Breast Surgery, The First Affiliated Hospital of Zhejiang Chinese Medical University, Hangzhou, Zhejiang, China.; 3 General Surgery, Cancer Center, Department of Gastrointestinal-Pancreatic Surgery, Zhejiang Provincial People's Hospital, Hangzhou Medical University, Hangzhou, Zhejiang, China.

**Keywords:** Ion channels, Cancer, Sensitization, Drug resistance.

## Abstract

Ion channels, as functional molecules that regulate the flow of ions across cell membranes, have emerged as a promising target in cancer therapy due to their pivotal roles in cell proliferation, metastasis, apoptosis, drug resistance, and so on. Recently, increasing evidence suggests that dysregulation of ion channels is a common characteristic of cancer cells, contributing to their survival and the resistance to conventional therapies. For example, the aberrant expression of sodium (Na^+^) and potassium ion (K^+^) channels is significantly correlated with the sensitivity of chemotherapy drugs. The endogenous calcium (Ca^2+^) channels contribute to the acquired resistance of osimertinib in epidermal growth factor receptor (EGFR) mutant non-small cell lung cancer cell lines. Ferrous ions (Fe^2+^) enhance the sensitivity of breast cancer cells to doxorubicin treatment. Preclinical models have also demonstrated the effect of specific ion channel blockers or modulators on anticancer drug resistance. This review describes the current understanding about the interaction between ion channels and the therapeutic efficacy of anticancer drugs. Then, the therapeutic potential of ion channel blockers or modulators in enhancing the sensitivity or overcoming the resistance of cancer cells to anticancer therapies is discussed. Targeting ion channels will hopefully offer a novel and promising strategy for overcoming cancer drug resistance.

## 1. Introduction

The asymmetric ion distribution across the cell membrane establishes a gradient fundamental to cellular function. Ion channels, a class of membrane-spanning proteins, provide selective conduits for specific ions, which orchestrate the delicate balance of ionic concentrations on either side of the membrane 1. These channels are pivotal in regulating transmembrane ion flux, maintaining cellular homeostasis, and propagating of signaling pathways essential for numerous biological processes including nerve impulse transmission, muscle contraction, and cancer cell signaling transduction 2-4. Ion channels play a crucial role in both the physiological and pathological processes of organisms, such as cell proliferation, migration, and apoptosis. Increasing researches have demonstrated that the expression of ion channels in cancer cells is frequently dysregulated. This abnormal expression and/or function can disrupt normal cellular processes, leading to the malignant transformation of normal cells. Consequently, this dysregulation is manifested as uncontrolled proliferation and spread, which are hallmark characteristics of cancer cells. The strong association between cancer hallmarkers and ion channel dysfunction leads us to classify cancer as a specific type of channel lesions, termed oncochannelopathies. Classic channelopathies often result from inherited mutations in ion channel genes, which alter the biophysical properties of the channel and then cause the disease. In contrast, oncochannelopathies often involve various malignancies in multiple ion channels, while channelopathies have traditionally been viewed as one channel diseases 1, 5.

Cancer remains the principal cause of mortality globally, significantly impeding the extension of life expectancy 6. The current therapeutic landscape for oncology is fraught with challenges, predominantly characterized by the emergence of drug resistance. This resistance confers a resilient phenotype upon many cancer cells, rendering them impervious to existing treatments 7. The ion channel-targeted therapy introduces a novel and potentially transformative strategy within the oncological domain. This paradigm has the capacity to surmount substantial challenges including the evolution of drug resistance, inequities in therapeutic delivery, and so on. Therefore, the strategic engagement of ion channels may enhance the therapeutic efficacy of cancer treatments and optimize the prognosis of cancer patients 8, 9.

Several ion channels exhibit differential expression in various malignancies and play pivotal roles in promoting malignant transformation through specific mechanisms. The combination of ion channel modulators with other anticancer drugs can generate synergistic effects, resulting in enhancing therapeutic efficacy or combating the drug resistance. This review systematically analyzes the interaction between the common ion channels (sodium, potassium, calcium, and chloride ion channels), trace element ion channels (iron, copper, zinc, and so on), and the therapeutic efficacy of anticancer drugs. The progress of ion channel blockers or modulators in augmenting the sensitivity of anticancer drugs or overcoming their resistance is also described. Our increasing understanding of ion channels in regulating therapeutic efficacy of anticancer drugs will hopefully offer a novel and promising strategy for combating cancer drug resistance.

## 2. The role of ion channels in cancer

Ion channels, govern the transmembrane movement of ions, are integral to maintaining cellular homeostasis. The role of ion channels in cancer is multifaceted and pivotal, influencing fundamental cellular processes in oncogenesis. The aberrant regulation of ion channels has been extensively documented across various cancer types (**Table 1**).

### 2.1 Sodium channel

In tumor regions, the concentration of sodium ions (Na^+^) is significantly higher than in normal tissues. This disparity is closely related to the formation and maintenance of the tumor immune microenvironment (TME), and interacts with the complex biological processes of tumorigenesis. Voltage-Gated Sodium Channels (VGSCs) contribute to an increase in Na^+^ influx, triggering a cascade of intracellular physiological reactions. These reactions include disruptions in Ca^2+^ concentration, pH balance, and overall cellular homeostasis 10. The SCNN1B gene encodes the essential β-subunit of the epithelial sodium channel (ENaC) complex. Elevated SCNN1B expression serves as an independent prognostic marker for prolonged survival in patients with advanced gastric cancer (GC). SCNN1B initiates the unfolded protein response (UPR) by degrading GRP78, which activates PERK, ATF4, XBP1s, and C/EBP homologous protein, ultimately leading to cancer cell apoptosis 11. In colorectal cancer (CRC), SCNN1B expression is significantly downregulated, indicating its role as a tumor suppressor. Experimental evidence shows that SCNN1B inhibits colon cancer cell proliferation and enhances apoptosis by regulating the c-Raf and MAPK signaling pathways 12. Increased expression of Nav1.5 has been demonstrated in oral squamous cell carcinoma (OSCC). Nav1.5 influences the proliferation, migration, and invasive capabilities of OSCC cells 13. So, sodium ion channels are closely related with carcinogenesis and cancer development.

### 2.2 Potassium channel

Potassium channels, the most diverse and well-studied ion channel family, play crucial roles in cell proliferation, apoptosis, cell volume regulation, and maintaining membrane potential. These functions make them a primary focus of cancer treatment 14, 15. Potassium channels are classified into four main groups: voltage-gated potassium channels, calcium-activated potassium channels, inwardly rectifying potassium channels, and two-pore domain potassium channels 16. KCNMA1 encodes the large-conductance calcium-activated potassium channel (BKCa), which acts as a pivotal tumor suppressor gene in carcinogenesis. Abnormal methylation of its promoter region modulates the expression of the key apoptotic gene PTK2 influencing the progression of GC 17. Activation of BKCa channels regulates the MEK/ERK pathway, impacting the development and progression of endometrial adenocarcinoma 18. Early studies show that inhibition of the Intermediate Conductance Calcium-Activated Potassium Channel 1 (IKCa1) potassium channel suppresses the proliferation of prostate cancer cells. This suppression might be related to IKCa1 activation-induced membrane potential hyperpolarization, which drives Ca^2+^ influx 19. Rapidly proliferating embryonic cells, stem cells, or cancer cells generally show a more depolarized state, cancer cells may exhibit this depolarized property to support their uncontrolled proliferation 20, 21. Elevated levels of KCa3.1 (KCNN4) have been identified across a spectrum of malignant neoplasms, including pancreatic cancer 22, breast cancer 23, non-small cell lung cancer(NSCLC) 24, and melanoma 25. KCa3.1 expressed on the mitochondrial inner membrane affects the survival of pancreatic ductal adenocarcinoma (PDAC) and melanoma cells, which is associated with mitochondrial function and intracellular calcium homeostasis 26.

The expression of Kv10.1 channels is upregulated in over 70% of cancers 27. In human breast adenocarcinoma exposure to the hERG1 channel activator NS1643 results in G0/G1 cell cycle arrest by activating of the senescence program, which is evidenced by increased protein levels of p21 and p16INK4a, and β-galactosidase activity 28. The potassium channel Kv1.3 is highly expressed in the mitochondria of various cancer cells and exists on the plasma membrane of different cell types. On the plasma membrane, Kv1.3 is involved in cell proliferation, while in the mitochondria, it plays a role in apoptosis in multiple types of tumor cells (mitochondrial channel) 29, 30. Caveolins regulate cell survival and participate in the plasma membrane targeting of Kv1.3. Specifically, the interaction between Kv1.3 and Cav1 in mitochondria, counteracts apoptosis, resulting in preventing Kv1.3-mediated cell death 29, 30. In osteosarcoma, silencing Kv1.5 inhibits cancer cell proliferation, induces G0/G1 cell cycle arrest, and promotes cell apoptosis 31. The ability to resist cell death is a hallmark of cancer, even under stressful conditions. Polycomb proteins (PcG) can regulate voltage-gated potassium channel genes in stem cells 32. PcG-dependent inhibition of the Kv1.5 channel gene KCNA5 contributes to cancer cell survival under stressful conditions 33. The activation of Kv11.1 channel increases the oxidative stress level, inhibits the NRF2-mediated antioxidant response mechanism, and enhances the lethal effect of the Kv11.1 channel activator on breast cancer cells 34. In GC, the gene encoding the voltage-gated potassium channel, KCNE2, is downregulated to contribute to the suppression of tumor proliferation, which may be associated with the downregulation of Cyclin D1 35.

Inwardly rectifying potassium channels, such as Kir2.2, enhance RelA phosphorylation and facilitate its cytoplasmic-to-nuclear translocation. This process activates the transcription factor NF-κB and upregulating its target genes including Cyclin D1, MMP9, and VEGF 36. Recent studies have demonstrated that the acid-sensing potassium channel KCNK3 impact various cancer types, such as prostate cancer 37, pancreatic cancer 38, hepatocellular carcinoma (HCC) 39, and NSCLC 40. Aberrant glucose metabolism stands as a defining characteristic of cancer 41. Overexpression of KCNK3 significantly inhibits the proliferative capacity and glycolytic processes of lung adenocarcinoma cells, which is associated with the activation of the AMPK-TXNIP pathway 42.

K^+^ transport is central to antitumor function and can be targeted to change T-cell exhaustion and augment cancer immunotherapy 43. The high K^+^ environment impacts the phosphorylation of the T-cell receptor (TCR)-mediated Akt-mTOR signaling pathway, independently of membrane potential changes. Overexpression of the Kv1.3 channel facilitates K^+^ efflux in T cells, thereby restoring T-cell function 44. Additionally, KCNAB2 is associated with immune infiltration defects. Its overexpression increases the expression of chemokines, among which CCL2 is crucial for immune cell recruitment 45. So, potassium channels may regulate immune cell functions within the TME, thereby enhancing their anti-tumor activity.

### 2.3 Calcium channel

Calcium, a ubiquitous and diffusible second messenger, plays a pivotal role in cell signaling mechanisms, orchestrating numerous fundamental physiological processes 46. The CACNG4 gene, encoding an L-type voltage-gated calcium channel γ subunit, influences tumor cell survival in breast cancer by closing channel pores, inhibiting Ca^2+^ influx, and altering crucial genes 47. Similarly, the CACNA1D gene, which encodes the CaV1.3 α1D subunit, is overexpressed in various cancers, including prostate, uterine, and colon cancer 48. The α1D subunit promotes the proliferation of endometrial cancer cells mediated by 17β-estradiol through the G protein-coupled estrogen receptor (GPER), leading to the phosphorylation of downstream molecules ERK1/2 and CREB 49. Additionally, CACNA1E enhances the proliferation of NSCLC cells by increasing current density and Ca^2+^ influx, activating the epidermal growth factor receptor (EGFR) signaling pathway 50. This gene is also associated with favorable histological outcomes in nephroblastoma recurrence 51.

Mitochondria are crucial in intracellular Ca^2+^ regulation and reactive oxygen species (ROS) generation 52. In colon cancer cells, RIPK1 interacts with the mitochondrial calcium uniporter (MCU), promoting proliferation by enhancing mitochondrial Ca^2+^ uptake and energy metabolism 53. MCU is regulated by MICU1, and its absence can lead to persistent mitochondrial calcium loading, excessive ROS production, and increased sensitivity to apoptotic stress 54. Metabolic dysregulations, particularly aerobic glycolysis, are implicated in tumor growth and chemoresistance 55, 56. In ovarian cancer, silencing MICU1 activates PDH by stimulating the PDPhosphatase-phosphoPDH-PDH axis, resulting in increased oxygen consumption, reduced lactate production, and inhibition of clonal growth of ovarian cancer cells 57.

In non-excitable cells, including most cancer cells, store-operated calcium entry (SOCE) serves as a crucial pathway for Ca^2+^ influx. SOCE activation are primarily involved in the interaction between stromal interaction molecule 1 (STIM1) and Orai1 58. STIM1 acts as a calcium sensor that triggers Ca^2+^ influx upon depletion of endoplasmic reticulum Ca^2+^ levels 59. Silencing STIM1 arrests the cervical cancer cell cycle at the S phase and G2/M phase 60. The molecular complexes in SOCE promote the proliferation, metabolism, migration, and invasion of GC cell by targeting MACC1 61. Knockdown of Orai3, a highly conserved paralog of Orai1 62, enhances SOCE in PDAC, leading to mitotic catastrophe and apoptosis in cancer cells 63.

Transient receptor potential canonical (TRPC) channels may be associated with both SOCE and non-capacitative calcium entry (NCCE) 64. Experimental evidence indicates that TRPC6 correlates with SOCE amplitude and the proliferation of HCC cells 65. In human follicular thyroid ML-1 cancer cells, TRPC1 plays a crucial role in the proliferation of thyroid cancer cells by regulating the expression of S1P3 and VEGFR2 in calcium-dependent mechanisms 66.

An imbalance between immune surveillance and tumor inflammation can lead to immunosuppression, ultimately resulting in tumor immune evasion 32. PD-L1 is a crucial checkpoint molecule in this context. By inhibiting the ORAI1 channel, intracellular calcium signaling is disrupted, hindering the release of PD-L1 from small extracellular vesicles (sEVs). This inhibition of PD-L1 release can suppress tumor growth and enhance systemic anti-tumor immunity. Ca^2+^-dependent proteins such as melanophilin and Synaptotagmin-like protein 2 are involved in the release of PD-L1 carried by sEVs 67. Therefore, calcium channel plays an important role in manipulating immune checkpoint blockade by PD-L1.

### 2.4 Other ion channels

Chloride ion channels play a crucial role in regulating the cell cycle and cell proliferation. These channels are often overexpressed in various tumors 68. ClC-3 upregulates Cyclin D1-CDK4/6 in nasopharyngeal carcinoma cells by inhibiting the expression of p21/p27, thereby affecting the cell cycle 69. ANO1, a calcium-activated chloride channel, is a significant oncogenic factor in the 11q13 amplification of breast cancer and other malignancies. It promotes breast cancer progression by activating the EGFR and CAMK signaling pathways 70. Conversely, overexpression of CLCA2 in nasopharyngeal carcinoma cells significantly reduces cell proliferation by inhibiting the FAK/ERK signaling pathway 71. Persistent acidosis is a prevalent characteristic of the TME across various cancer types, including glioblastoma multiforme (GBM) 72. ASIC1a, an acid-sensing ion channel, is highly sensitive to extracellular protons. Acidosis triggers RIPK1-dependent death in glioblastoma stem cells via the activation of ASIC1a 73.

Ion channels that transport trace elements, alongside common ion channels like sodium, potassium, and calcium, are crucial for maintaining cellular stability and contributing to cancer development. Iron is essential for DNA synthesis, cellular metabolism, and proliferation 74. Transferrin receptor (TFR) is overexpressed in many types of cancers, such as liver cancer 75, kidney cance 76, glioma 77, and pancreatic cancer 78, compared to non-tumor tissues, which makes it a promising target for cancer therapy. Excess cellular iron is toxic, recent studies have found that OTUD1 acts as a deubiquitinating enzyme of iron-responsive element-binding protein 2 (IREB2), which can block its degradation, thereby promote the expression of transferrin receptor protein 1 (TFRC) and enhance cellular iron uptake, leading to increased intracellular ROS production and ferroptosis in CRC 79. In addition to increasing iron absorption and reducing iron storage, cancer cells also reduce iron export 80. MCOLN1/ mucolipin TRP channel 1 (TRPML1), a non-selective cation channel localized in lysosomes, functions as a Fe^2+^ permeable channel in late endosomes and lysosomes 81, and is involved in vesicle fusion and fission processes 82. TRPML1 can attenuate MAPK and mTORC1 signaling, maintain protein homeostasis and facilitate macropinocytosis, thereby promoting the survival and proliferation of melanoma cells 83. The iron exporter ferroportin 1 (FPN1), the only known iron exporter in vertebrates, plays a crucial role in myeloma. Restoration of FPN1 expression is shown to decrease intracellular liable iron pool, inhibit STAT3-MCL-1 signaling, which in turn suppresses myeloma cell proliferation 84. Additionally, cancer cells upregulate autophagy processes to meet their nutritional needs. MCOLN1/TRPML1, a key player in autophagy, is implicated in various cancers by promoting carcinogenic autophagy. TRPML1 serves as a crucial ion channel that mediates the release of metal ions from lysosomes 85. Besides mediating Ca^2+^ influx, MCOLN1 also facilitates lysosomal zinc (Zn^2+^) inflow. This action blocks the interaction between STX17 and VAMP8, thereby regulating carcinogenic autophagy 86. Zn^2+^ is an essential trace element necessary for cell function. However, excessive Zn^2+^ release can impair mitochondrial functions, particularly the electron transport chain, leading to energy depletion and cell death. In metastatic melanoma, the protein TRPML1 is upregulated 87. Activation of TRPML1, rather than its inhibition, induces cell death. Specifically, activation of ML-SAs (presumably TRPML1 activators) results in lysosomal Zn^2+^-dependent necrotic cell death 88. Copper also plays a crucial role in metabolic homeostasis. The E3 ubiquitin ligase Nedd4l inhibits the expression of Copper Transporter 1 (CTR1) through ubiquitination. This Nedd4l-CTR1 signaling pathway modulates AKT kinase activity in a copper-PDK1 binding-dependent manner. Therefore, targeting the CTR1-copper pathway to counteract hyperactive AKT-driven cancers represents a potential therapeutic strategy 89. So, Ion channels that transport trace elements influence cellular physiological functions by regulating the balance and transport of trace ions, thereby promoting or inhibiting cancer development.

**Figure 1:**
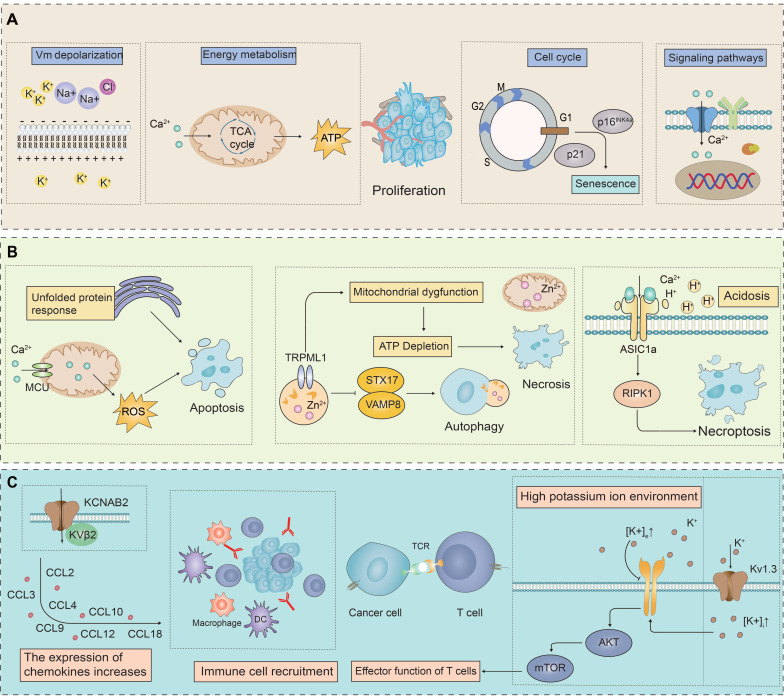
** The role of ion channels in the cell proliferation, death and tumor immune microenvironment (TME).** Compared to normal cells, ion channels in cancer cells often show abnormal expression patterns, which impact cell proliferation, death resistance, and the remodeling of the TME.** (A)** Ion channels significantly influence tumor cell proliferation by regulating cell membrane potential, energy metabolism, cell cycle, and intracellular Ca^2+^ concentration These processes modulate signaling pathways that are critical for cancer cell proliferation. **(B)** By activating certain ion channels, it interferes with the physiological processes of cancer cells, leading to cell death. Ion channels induce apoptosis in cancer cells by generating excessive ROS through the endoplasmic reticulum's UPR and by modulating mitochondrial Ca^2+^ concentrations. Additionally, ion channels affect mitochondrial function by altering Zn^2+^ concentrations, leading to ATP depletion and necrotic cell death. They also regulate autophagy by mediating the release of Zn^2+^ from lysosomes into the cytosol. Furthermore, acidosis in the TME induces RIPK1-dependent necroptosis via acid-sensing ion channels. **(C)** Ion channels, particularly potassium ion channels, play a crucial role in tumor immunity. They influence the TME and the function of immune cells through various mechanisms.

**Figure 2:**
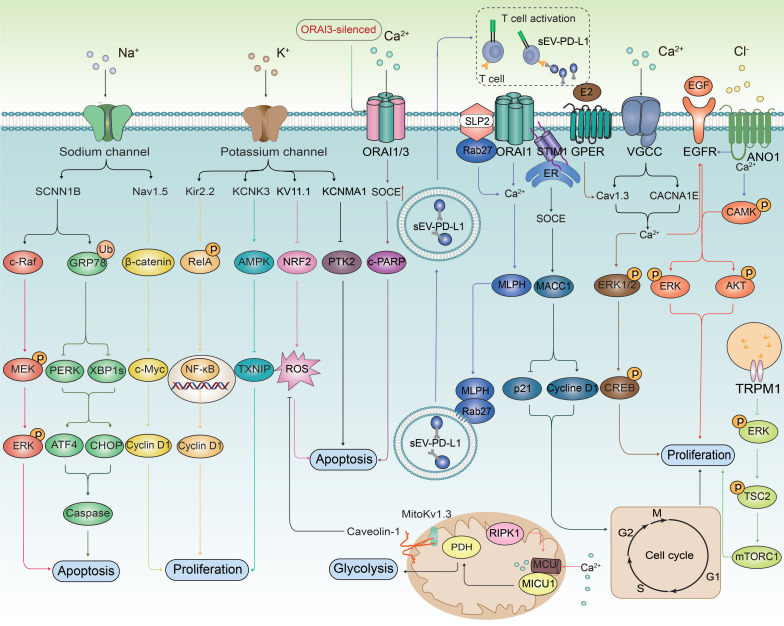
** Ion channels trigger key biological effect on cancer cells by regulating multiple signaling pathways.** Ion channels play a crucial role in regulating the biological behavior of cancer cells. They influence the proliferative ability of cancer cells by modulating multiple signal transduction pathways, including NF-κB, β-catenin, AMPK, MACC1, MAPK/ERK, Ca^2+^/CaMK, and PI3K/Akt. Furthermore, ion channels are involved in the regulation of apoptosis through pathways such as MAPK, endoplasmic reticulum stress, oxidative stress signaling (NRF2), PTK2, PARP, and apoptosis-related proteins. In the context of tumor immune escape mechanisms, ion channels enhance the immune evasion capabilities of tumor cells by regulating the release of programmed death PD-L1 in sEV. Mitochondrial ion channels influence the activity of pyruvate dehydrogenase (PDH), thereby affecting the glycolysis process, which provides energy for cancer cells and promotes their survival.

In summary, the biological role of ion channels in tumor cells is both multi-dimensional and complex, with their abnormal expression being a critical phenomenon in tumorigenesis. This abnormal expression is intricately involved in the proliferation and death resistance of tumor cells as well as the reconstruction of the TME. Specifically, in tumor cells, the expression levels of certain ion channels are abnormally elevated, which may promote the proliferation and survival of these cells. These ion channels regulate the membrane potential by controlling the concentrations of K^+^ and Na^+^ ions inside and outside the cell, thereby influencing the proliferative state of the tumor cells. In mitochondria, the flow of Ca^2+^ can induce membrane depolarization, promote ATP synthesis, and thus enhance cell proliferation (**Figure 1A**). Conversely, some studies have found that the expression of certain ion channels in tumor cells is significantly down-regulated to resist programmed cell death, potentially acting as tumor suppressors. Activation or ectopic expression of these ion channels can induce programmed cell death in cancer cells (**Figure 1B**). The activation of potassium ion channels can activate AKT/mTOR signaling pathway, enhance T cell function, and promote the infiltration of immune cells by increasing the expression of chemokines, thereby enhancing the immune response to tumors (**Figure 1C**). Ion channels, as key membrane proteins, exhibit highly selective permeability and regulate the transmembrane transport of specific ions. Through this interaction, they significantly influence intracellular signaling pathways. These channels are deeply involved in the pathophysiological characteristics of various cancer markers, affecting them to varying degrees through a multitude of distinct signaling mechanisms, as shown in **Figure 2**. These findings provide a theoretical basis for developing novel anti-cancer therapeutic strategies targeting ion channels.

## 3. Targeting ion channels to overcome cancer resistance

During cancer treatment, the issue of reduced sensitivity and drug resistance due to cellular evolution has emerged as a significant barrier to therapeutic efficacy. Ion channel modulators will hopefully offer a promising strategy to enhance the sensitivity or reverse the resistance of anticancer drugs (**Table 2**).

### 3.1 Ion channels and chemotherapy

#### 3.1.1 Enhancing anticancer drug sensitivity

In the field of cancer therapy, enhancing the sensitivity of tumor cells to therapeutic interventions is a pivotal strategy for improving overall treatment efficacy. The KCNG1 gene expression is significantly higher in triple-negative breast cancer (TNBC) compared to other breast cancer subtypes. This elevated expression is positively correlated with increased sensitivity to chemotherapy drugs such as cisplatin and oxaliplatin, suggesting that targeting KCNG1 with its inhibitor guanidine hydrochloride (GuHCl) may be an effective treatment strategy for TNBC 90. The dual-action drug liluzole, combined with a KCa3.1 activator and a Kv11.1 inhibitor, significantly enhanced cisplatin uptake. This combination synergistically increased cisplatin-induced apoptosis and anti-proliferative effects in CRC cells 91, 92. The combination of the IK1 channel activator 1-EBIO with cisplatin has been demonstrated to enhance caspase-3/7 activity, thereby augmenting the apoptotic cell death induced by cisplatin. This finding underscores the potential of IK1 channel activation in improving the efficacy of cisplatin treatment 93. Additionally, other research has identified a significant relationship between cell volume regulation and cisplatin sensitivity, highlighting the role of the VSOR Cl^-^ channel, which exhibits a similar mode of action 94. In parallel, the combination of sparfloxacin (SPFX), a HERG K^+^ channel blocker, with 5-fluorouracil has been shown to synergistically inhibit the proliferation and induce apoptosis of colon cells 95. The Nav1.5 activator veratridine has been found to increase 5-FU-induced apoptosis in CRC cells, thereby enhancing the sensitivity of chemotherapy 96. Calcium channel blockers (CCBs) such as lercanidipine and amlodipine have been shown to inhibit YY1/ERK/TGF-β-mediated transcription, thereby increasing the sensitivity of GC cells to doxorubicin. The potential use of these blockers in targeted and combined therapies for GC is suggested by recent finding 97. Similarly, the combined use of mibefradil, an inhibitor of T-type calcium channels, with carboplatin has been found to synergistically enhance apoptosis in ovarian cancer cells *in vitro*. Mibefradil achieves this by reducing AKT phosphorylation, increasing the levels and nuclear retention of FOXO transcription factors, and suppressing the expression of the anti-apoptotic gene BIRC5 98. In liver cancer, increased expression of Orai1 is observed. Inhibition of Orai1-mediated Ca^2+^ influx, known as store-operated Ca^2+^ entry (SOCE), significantly enhances the sensitivity of the human liver cancer cell line HepG2 to 5-FU. This is accomplished by potentiating the inhibition of the PI3K/AKT/mTOR pathway and promoting autophagic cell death induced by 5-FU 99. Cancer stem cells (CSCs), a small subset of tumor cells with stem cell characteristics, have been implicated in the resistance to chemotherapy, potentially leading to tumor rebound and recurrence 100, 101. In epithelial ovarian cancer, the resistance is linked to the presence of CSCs. From a library of FDA-approved compounds, four CCBs were identified for their ability to disrupt the characteristics of ovarian CSCs by inhibiting the AKT and ERK signaling pathways and inducing apoptosis. When combined with cisplatin, these CCBs synergistically suppress the activity and proliferation of ovarian CSCs 102. In NSCLC cells, cisplatin treatment leads to the upregulation of Orai3 and CSC markers. This process involves the modulation of Ca^2+^ influx, which increases the expression of CSC markers Nanog and SOX-2 via the PI3K/AKT pathway. Silencing Orai3 or altering extracellular Ca^2+^ levels has been shown to enhance sensitivity to cisplatin 103. Furthermore, the voltage-dependent calcium channel α2δ1 subunit has been proposed as a potential marker for GC stem cells. The knockdown of α2δ1 significantly diminishes the spherogenesis and tumorigenic capacity of these stem cells, while concurrently increasing the sensitivity of cisplatin *in vitro* 104. The small-cell lung cancer (SCLC) cells expressing the α2δ1 subunit exhibit cancer stem cell-like characteristics, which may contribute to chemotherapy resistance. The 1B50-1 antibody, a monoclonal antibody specifically targeting the α2δ1 subunit, is observed to improve the efficacy of chemotherapy and delay relapse, particularly in cases with a relatively low proportion of α2δ1^+^ SCLC cells. 105. The inhibition of transient receptor potential melastatin-2 (TRPM2), an important regulator of Ca^2+^ influx, has been shown to induce cell death in several malignancies, including T-cell leukemia 106. In neuroblastoma, TRPM2 knockout results in reduced tumor proliferation and increased sensitivity to doxorubicin, which are associated with Ca^2+^ activity 107. Similarly, TRPM2 is highly expressed in acute myeloid leukemia (AML), and its knockout leads to inhibit cell proliferation and heighten the sensitivity to adriamycin. This effect is attributed to impaired mitochondrial function, disrupted autophagy, and elevated ROS levels 108. TRPM8, another calcium-permeable cation channel, enhances epirubicin-induced apoptosis when its expression is knocked down, primarily by reducing phosphorylated p44/p42 levels and promoting JNK activation triggered by epirubicin 109. Autophagy plays an important role in cell survival and chemotherapy sensitivity 110-112. TMEM16A ion channel is responsible for calcium-activated chloride transport in epithelial tissues and its overexpression is considered to be associated with cisplatin resistance by promoting lysosomal flux in squamous cell carcinoma of the head and neck (SCCHN). Notably, the lysosomal inhibitor hydroxychloroquine (HCQ) has been demonstrated to synergistically enhance the cytotoxic effects of cisplatin on SCCHN cells *in vitro* 113. ClC5, a member of the chloride channel family, contributes to the chemoresistance of multiple myeloma cells to bortezomib (BZ) treatment by promoting pro-survival autophagy. Interestingly, the knockout of ClC5 increases the sensitivity of these cells to BZ, highlighting its potential as a therapeutic target 114. The transport mechanisms of platinum-based drugs involve various drug transporters, such as volume-regulated anion channels (VRACs). In head and neck cancer cells, the expression level of VRAC is crucial for determining the responsiveness of platinum drugs 115. The substrate selectivity of the VRAC channel is determined by the composition of LRRC8 subunits, which regulate the intracellular uptake of cisplatin and carboplatin and promote apoptosis. Specifically, the LRRC8D subunit is essential for maintaining cell volume homeostasis and may significantly influence tumor responsiveness to cisplatin/carboplatin 116.

Ion channels also play a crucial role in regulating trace element transport and influencing cancer cell sensitivity to chemotherapy. Iron, for instance, is implicated in promoting ovarian cancer through its absorption via transferrin receptor TFRC. Iron facilitates DNA damage repair through the FTH1/FTL/POLQ/RAD51 pathway, and iron chelators enhance the sensitivity of ovarian cancer to carboplatin 117. In mammals, TFR1 mediates the endocytosis of transferrin-bound iron from the extracellular environment, while divalent metal transporter 1 (DMT1) translocates iron ions from endocytosomes into the cytoplasm 118. DMT1 is antagonized by GSK-3β, which manipulates iron-induced cell death, offering insights into potential chemotherapy targets 119. Iron metabolism exhibits a dual role in tumor development, both promoting and inhibiting it. Iron catalyzes the conversion of hydrogen peroxide into ROS, and excess ROS can trigger lipid peroxidation, leading to ferroptosis when the antioxidant system is overwhelmed 120. TRPML1 regulates lysosomal iron release into the cytoplasm, and its inhibition promotes ferroptosis in breast CSCs to reduce their stemness and enhance the sensitivity of breast cancer cells to doxorubicin 121. Intracellular Fe^2+^ is exported by the membrane protein FPN1 and lncRNA MAF transcription factor G antisense RNA 1 (MAFG-AS1) in bladder urothelial carcinoma (BUC) cells. Inhibition of MAFG-AS1 expression increases cisplatin sensitivity in BUC cells by promoting ferroptosis 122. Zinc, another essential trace element, is involved in DNA synthesis, enzyme activity, and nucleic acid metabolism 123. ZIP4, a regulator of intracellular zinc, inhibits the gemcitabine transporter ENT1, thereby reducing the sensitivity of pancreatic cancer cells to gemcitabine 124. Copper ions, when in excess, lead to the endocytosis and degradation of CTR1 125, 126, resulting in the decrease of cisplatin uptake. In a mouse model of human cervical cancer, combining copper chelators with cisplatin enhances the therapeutic efficacy of cisplatin 127. These results are summarized in **Table 2**.

#### 3.1.2 Reversing anticancer drug resistance

Ion channels are also pivotal in the development of drug resistance in tumor cells. Multidrug resistance (MDR) is a primary cause of chemotherapy failure, responsible for up to 90% of cases, making it crucial to combat tumor resistance to enhance the efficacy of anticancer therapy 128. A promising approach to counteract this resistance is targeting ion channels to reverse the resistance of tumor cells to anticancer drugs. In SCLC, for instance, the KCNJ2/Kir2.1 channel is expressed in 44.23% of tissues and influences cell growth and drug resistance by regulating the expression of MDR protein 1 (MRP1/ABCC1). This channel is modulated by the Ras/MAPK pathway and miR-7, exhibiting its potential as both a prognostic biomarker and a therapeutic target for overcoming chemotherapy resistance in SCLC 129. Additionally, in AML, the long non-coding RNA potassium voltage-gated channel subfamily Q member 1 overlapping transcript 1 (KCNQ1OT1) shows elevated expression levels in the cells resistant to the chemotherapy drug adriamycin (ADR). Targeting the KCNQ1OT1/miR-193a-3p/Tspan3 axis presents a potential therapeutic strategy for overcoming chemoresistance in AML 130. The calcium/calmodulin signaling pathway is particularly active in gemcitabine-resistant tumor cell subsets in PDAC. Calcium channel blockers (CCBs) have demonstrated their ability to inhibit survival-promoting ERK signaling *in vitro*, significantly enhancing the therapeutic efficacy of gemcitabine in both orthotopic xenograft and transgenic PDAC models 131. Furthermore, CCBs have been shown to reverse MDR induced by docetaxel and vincristine in NSCLC cells 132, 133. The combination therapy of nifedipine (a dihydropyridine class CCB) and cisplatin has been found to synergistically inhibit tumor cell proliferation and primary tumor growth both *in vitro* and *in vivo*, inducing apoptosis in cisplatin-resistant human glioblastoma cells 134. Autophagy, known as a pro-survival signal, can contribute to the chemoresistance 135-137. In temozolomide-resistant GBM models, the knockdown of Ca_v_3.1 calcium channel reduced GBM cell viability and slowed tumor progression, which was associated with the transcriptional downregulation of p62/SQSTM1 and defects in autophagy 138. In breast cancer, the overexpression of Orai3 calcium channel plays a critical role in promoting cell growth and survival, thereby conferring resistance to chemotherapeutic drugs. Mechanistically, this process involves the downregulation of the p53 tumor suppressor protein, which is mediated through the pro-survival PI3K/Sgk-1/Akt-1 signaling pathway. The degradation of p53 is further associated with the actions of Mdm2 and Nedd4-2, which are key regulators in this pathway 139. Additionally, inhibitor of apoptosis-stimulating protein of p53 (iASPP) maintains intracellular Ca^2+^ balance by preventing Gp78-mediated degradation of Ca^2+^-channel protein transmembrane and coiled-coil domains 1 (TMCO1), thereby inhibiting tumor cell growth and overcoming the resistance to therapeutic drugs in colon cancer 140. EGFR overactivation is associated with resistance to various therapies, including chemotherapy, radiotherapy, and immunotherapy 141, 142. In cervical cancer, TRPV1 promotes autophagy-mediated EGF secretion via Ca^2+^ influx, which induces the acquisition of cisplatin resistance. TRPV1 inhibition using a small-molecule agent AMG9810 can effectively overcome cisplatin resistance in cervical cancer cells 143. Disruption of intracellular Ca^2+^ balance is linked to cellular escape from death 144, 145, and abnormal expression of calcium regulatory genes may confer cisplatin resistance. Tranilast in combination with cisplatin significantly enhances apoptosis and reverses drug resistance by inhibiting TRPV2 channels and preventing the efflux of Ca^2+^ ions 146. In breast cancer, doxorubicin up-regulates TRPC5 expression and promotes autophagy through the CaMKKβ/AMPKα/mTOR signaling pathway. Silencing TRPC5 and inhibiting autophagy can reverse the resistance of breast cancer cell to adriamycin 147.

Lysosomes play a critical role in promoting the sequestration of drugs, making them a promising target for overcoming chemical resistance. Modulating TRPML1-mediated lysosomal exocytosis can regulate cisplatin resistance. This regulation is associated with altered metabolomic signatures due to TRPML1 inhibition 148. Changes in cellular metabolism can contribute to the development of resistance to chemotherapeutic drugs 149. In recent years, the role of chloride channel-3 (ClC-3) in tumor drug resistance mechanisms has gained significant attention. In human lung adenocarcinoma and breast cancer cell lines, ClC-3 is highly expressed, inducing MDR through the activation of the NF-κB signaling pathway and the up-regulation of P-gp expression 150. Furthermore, ClC-3 is crucial in mediating cisplatin resistance in human erythroleukemia cells, glioma cells, and cholangiocarcinoma cells 151-153. This highlights ClC-3 as a potential target for overcoming chemotherapy resistance in cancer treatment. In paclitaxel-resistant ovarian cancer cells, ClC-3 overexpression enhances the interaction with β-tubulin, thereby increasing drug resistance. Notably, silencing ClC-3 partially restores the sensitivity of these cancer cells to paclitaxel, underscoring its potential as a therapeutic target 154. Numerous studies have demonstrated a close relationship between exosomes and chemotherapy resistance across various cancers. In the context of GC cells, vincristine-resistant cell lines exhibit elevated levels of chloride intracellular channel 1 (CLIC1) expression. This resistance is potentially linked to the up-regulation of P-gp and Bcl-2, facilitated by exosome-mediated CLIC1 transfer, which induces vincristine resistance *in vitro*. Notably, silencing CLIC1 expression significantly reduces the semi-inhibitory concentration (IC_50_) of vincristine 155. Furthermore, CLIC1 is associated with drug resistance in human choriocarcinoma 156. Changes in extracellular pH homeostasis are prevalent in most solid tumors, influencing cancer proliferation and migration. Acid sensing ion channel 1a (ASIC1a), a H^+^-gated cation channel, plays a significant role in these processes 157, 158. Notably, ASIC1a is overexpressed in drug-resistant HCC cells and is implicated in drug resistance through the Ca^2+^/PI3K/AKT pathway. ASIC1a knockout can overcome drug resistance in HCC cells 159. EMT induced by the tumor microenvironment is closely related to tumor invasion and drug resistance. The inactivation of ASIC1a has been shown to suppress cell migration and invasion. This mechanism is specifically regulated by the AKT/GSK-3β/Snail pathway, driven by the TGFβ/Smad signal, which modulates the expression of α-catenin, β-catenin, vimentin, and fibronectin 160.

Ferroptosis, a process involving TFRC, is closely linked to tumor cells and plays a significant role in breast cancer drug resistance. The upregulation of TFRC in ADR-resistant breast cancer cells can activate ferroptosis, thereby reversing ADR resistance 161. Zinc transporters in the cell membrane, including the Znt/SLC30 family (responsible for Zn efflux) and the ZIP/SLC39 family (absorbing Zn from the extracellular environment), are crucial in this context 162. Specifically, the expression of ZIP10 influences the sensitivity of cancer cells to chemotherapeutic drugs and affects the clinical outcomes of osteosarcoma. Knocking out ZIP10 inhibits osteosarcoma cell proliferation and impairs the chemoresistance through the ZIP10-ITGA10-PI3K/AKT axis 163. The copper transporter CTR1 affects the influx of platinum drugs into cells. Lower CTR1 levels are generally associated with increased cisplatin resistance in tumors 164, while higher CTR1 expression in NSCLC patients is associated with higher survival rates 165. Consequently, manipulating CTR1 with copper-chelating drugs can selectively reverse resistance to platinum treatment 166. In human cervical cancer oxaliplatin-resistant S3 cells, the copper chelator D-penicillamine increases the therapeutic efficacy of platinum drugs in oxaliplatin resistant tumors 167. Therefore, targeted ion channel therapies have demonstrated significant potential in overcoming chemotherapeutic drug resistance (**Table 2**).

### 3.2 Ion channels and targeted therapy

The development of resistance to targeted cancer therapies presents an increasingly formidable clinical challenge. Tumor cells exhibit resistance to these drug treatments through intricate molecular adaptive changes, which limits the long-term efficacy of precision medicine. Ion channels are emerging as important tumor targets and potential cancer biomarkers 168. Primary chronic lymphocytic leukemia (CLL) cells from ibrutinib-resistant patients can be killed with Kv1.3 potassium channel inhibitor PAPTP. Thus, PAPTP emerges as a potential alternative therapeutic option for CLL 169. For patients with BRAF-mutant melanoma, the primary and acquired resistance of BRAF and MEK inhibitors remain major treatment challenges 170. The combination of proteasome and Kv1.3 channel inhibitors results in synergistic effects and prevents the outgrowth of both drug-resistant and -sensitive BRAF-mutant melanoma cells 171. TRAM-34, a specific inhibitor of the KCa3.1 channel 172, is found to significantly enhance the induction of apoptosis by vemurafenib in melanoma cells 173. NSCLC treatment also faces challenges, particularly with the development of resistance to EGFR tyrosine kinase inhibitors (TKIs) after initial efficacy 174. Recent studies indicate that combining the blockade of the KCa3.1 channel with the EGFR TKI erlotinib can effectively enhance tumor cell responsiveness to erlotinib 175. Clinically, the use of BRAF inhibitors, MEK inhibitors, or a combination of both has been proven to significantly extend the progression-free survival and overall survival of patients with malignant melanoma 176, 177. However, resistance to these treatments remains a significant issue. Inhibiting T-type calcium channels has been found to induce differentiation and death of drug-resistant melanoma cells *in vitro* and reverse the resistance to MAPK inhibitors *in vivo* 178. The melanoma patients with BRAF^V600E^ mutation can be treated with kinase inhibitors, such as vemurafenib, but these patients frequently develop the acquire resistance to these drugs. T-type calcium channel (TTCC) blocker mibefradil can induce apoptosis via autophagy inhibition in drug-resistant BRAF melanoma cells, which provides a therapeutic strategy toward BRAF inhibitor resistance 179. The elevated TRPM2 expression was observed in EGFR mutant non-small cell lung cancer cell lines treated with osimertinib. The knockdown of TRPM2 inhibits the endogenous flow of Ca²^+^, thereby enhancing the apoptotic effect of osimertinib *in vitro* and *in vivo*. Therefore, targeting TRPM2 is expected to be a promising strategy for overcoming and preventing osimertinib acquired resistance 180. The intracellular Cl^-^ channel protein ClC-3 contributes to the resistance of cancer cells to chemotherapy drugs and HER2-targeted therapies 181. The intracellular Cl^-^ regulation by ANO1/ClC-3 is closely related to the transcription of the HER2 gene in HER2-positive breast cancer cells. Consequently, inhibitors of ANO1/ClC-3 may represent an effective therapeutic strategy for patients with resistance to anti-HER2 therapies 182. Additionally, the inhibition of TMEM16A/ANO1, a Ca^2+^-activated Cl^-^ channel, increases the sensitivity to EGFR and HER2/ERBB2 targeted therapies 183. In summary, targeting ion channels presents a promising strategy to overcome resistance in various cancers, including CLL, melanoma, and NSCLC. Further researches into these mechanisms may lead to more effective and durable anticancer therapies.

### 3.3 Ion channels and immunotherapy

Ion channel modulators can enhance the immune system's attack on tumors through various mechanisms, thereby improving therapeutic efficacy of immunotherapy. Nifedipine (NIFE), a calcium channel blocker, inhibits the expression of programmed death-ligand 1 (PD-L1) on CRC cells and programmed death-1 (PD-1) on CD8^+^ T cells. This finding reveals the significant role of calcium channel-related signaling pathways in PD-1/PD-L1-mediated tumor immune escape. In the further study, the researchers show that NIFE is more effective against tumors when used in combination with anti-PD-1 antibodies. This combination therapy suggests a potential synergistic effect, offering a more effective strategy for enhancing PD-1-based antitumor immunotherapy 184. TRPML1-mediated ferroptosis plays a critical role in AKT-driven tumorigenesis and anticancer drug resistance. The inhibition of TRPML1 can suppress AKT-driven tumorigenesis and enhance the sensitivity of tumor cells to ferroptosis induction therapy, radiation therapy, and immunotherapy. Specifically, WT-iRGD, a peptide targeting TRPML1, inhibits tumorigenesis and promotes cancer therapy by blocking the interaction between TRPML1 and ARL8B. This blockade leads to increased lipid peroxidation levels and the activation of cytotoxic CD4^+^ and CD8^+^ T cells in mice, particularly when combined with PD1 antibodies 185. The P2RX7 receptor (also known as P2X7R) is an ATP-gated ion channel, primarily presents in immune cells and some tumor cells 186. P2RX7 has been demonstrated to play a crucial role in the maturation of macrophages and dendritic cells (DCs), as well as in the secretion of the proinflammatory cytokines IL-1β and IL-18. Moreover, P2RX7 coordinates immunogenic cell death (ICD) and enhances the ability of DCs to activate and present tumor antigens to T cells, positioning it as a positive regulator of the anti-tumor immune response 187. In immune cells, P2RX7 negatively regulates the number and function of regulatory T cells (TREGs), thereby inhibiting their immunosuppressive activity. This regulatory function is particularly relevant in the treatment of NSCLC, where only a subset of patients responds to immunotherapy. The P2RX7 activator, HEI3090, has been shown to augment an anti-tumor immune response, when used in combination with PD-1 immune checkpoint inhibitor. Mechanistically, the activation of P2RX7 leads to an increase in IL-18 production in an NLRP3-dependent manner, which subsequently activates NK cells and CD4^+^ T cells to produce IFN-γ, thereby enhancing tumor immunogenicity 188.

**Figure 3:**
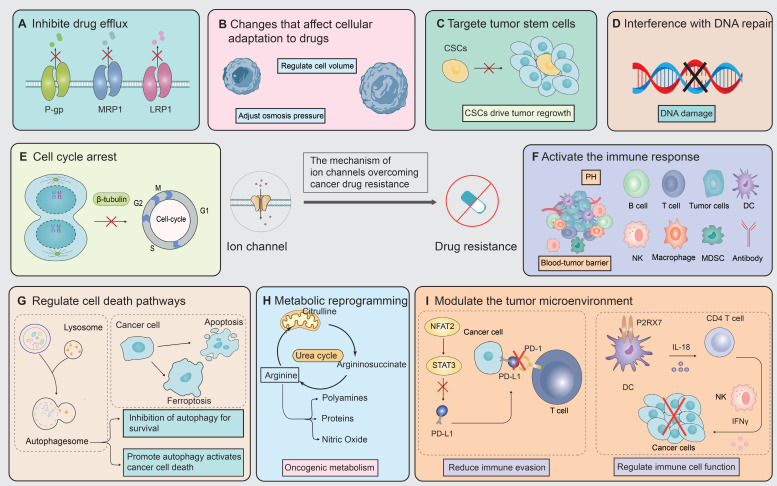
** Therapeutic mechanisms targeting ion channels to overcome cancer drug resistance.** Targeting ion channels is a therapeutic strategy to overcome anti-cancer resistance and involves multiple pathways. **(A)** Firstly, they influence efflux pumps, inhibiting drug efflux and thereby increasing intracellular drug concentration. **(B)** Modulating cellular adaptive changes to chemotherapy drugs, such as osmoregulation, which affects cell volume. **(C)** Targeting specific markers or signaling pathways of tumor stem cells to reduce tumor resistance. **(D)** Interfering with DNA repair pathways to reduce cancer cells' ability to repair DNA damage induced by chemotherapy. **(E)** modulating cyclins or arresting the cell cycle by inhibiting mitosis. **(F)** Promoting drug efficacy by altering the composition of immune cells, PH values, and the blood-brain barrier within the TME. **(G)** Enhancing the killing effect of chemotherapeutic drugs by promoting apoptosis, autophagy, ferroptosis, and other cell death pathways. **(H)** Modulating tumor metabolic pathways to reduce resistance to chemotherapy drugs. **(I)** Regulating the immune system by reducing immune evasion and influencing the function of immune cells.

**Figure 4:**
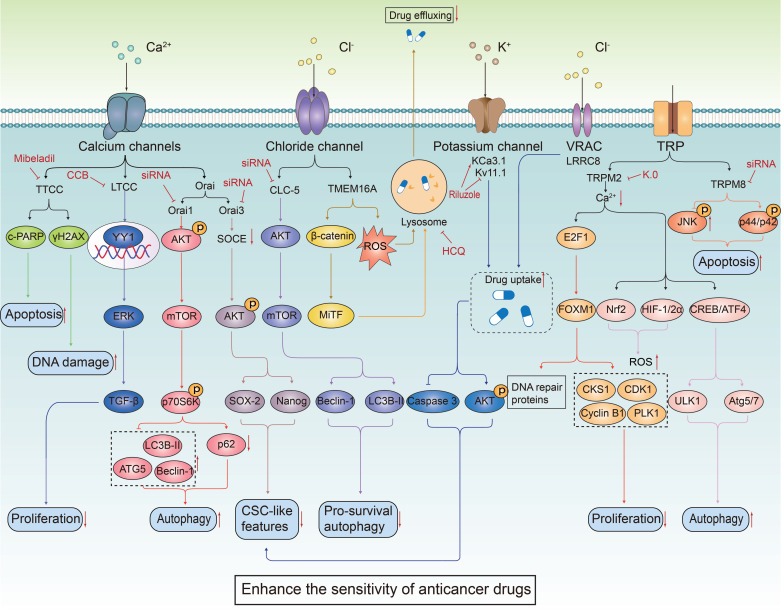
** The combined use of ion channel-targeting agents with anticancer drugs enhances their sensitivity through related signaling pathways.** Given the close relationship between ion channels and cancer, drugs targeting ion channels, when used in combination with anticancer drugs, exhibit synergistical effect on tumor cells. By precisely regulating the ion balance within these cells, they effectively inhibit abnormal proliferation. This regulation significantly enhances apoptosis and autophagy processes while exacerbating DNA damage. These combined effects not only undermine the fundamental survival mechanisms of tumor cells but also markedly increase their sensitivity to traditional anticancer drugs.

**Figure 5:**
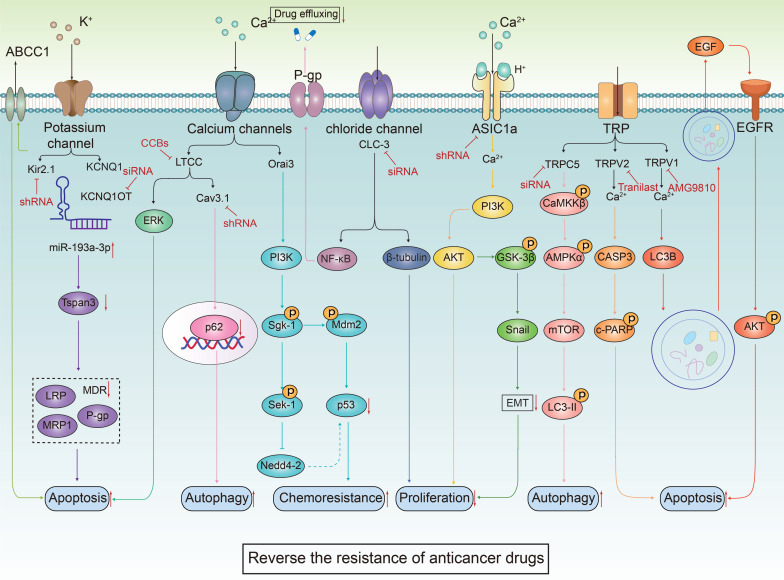
** The combined use of ion channel-targeting agents with anticancer drugs reverses the resistance via multiple signaling pathways.** The combination of ion channel targeting drugs and anticancer drugs can effectively inhibit the abnormal proliferation of tumor cells and significantly enhance the process of apoptosis and autophagy by regulating ion homeostasis and related signaling pathways. This combination therapy especially targets tumor cells that have shown resistance to conventional anticancer drugs, showing a significant resistance reversal effect. In addition, abnormal function of specific ion channels such as Orai3 is directly related to the development of chemotherapy resistance in tumor cells. This resistance occurs because the degradation of p53 reduces the cell's response to chemotherapeutic-induced apoptosis.

**Figure 6:**
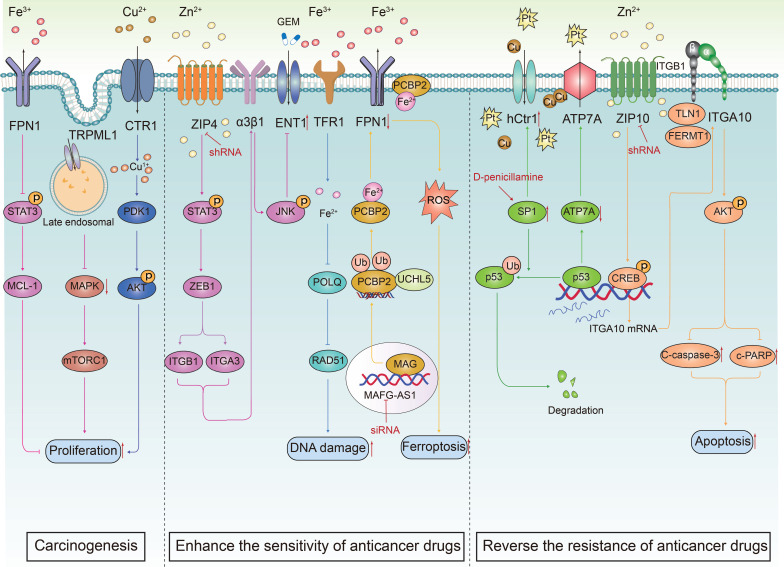
** The transport trace elements are involved in carcinogenesis and anticancer drug resistance.** Metal ions, including Fe, Zn and Cu, interact with ion channels on the cell membrane, thereby regulating intracellular ion concentrations. This regulation plays a crucial role in fine-tuning cell signal transduction pathways, which in turn influences key biological processes such as cell proliferation and apoptosis. It is worth noting that the combination of ion channel modulators transporting metal elements and anticancer drugs can inhibit drug efflux, promote cell apoptosis, increase DNA damage, cause iron sag, effectively improve the sensitivity of anticancer treatment and reverse drug resistance of cancer cells.

The ion channel regulators, when combined with conventional cancer therapies, can combat anticancer drug resistance through multiple mechanisms. These mechanisms include inhibiting drug efflux (**Figure 3A**), regulating cell volume changes (**Figure 3B**), targeting tumor stem cells (**Figure 3C**), interfering with DNA repair mechanisms (**Figure 3D**), inhibiting cell proliferation processes (**Figure 3E**), activating immune responses (**Figure 3F**), promoting cell death (**Figure 3G**), inhibiting metabolic reprogramming (**Figure 3H**), and regulating tumor microenvironment (**Figure 3l**). The combination of ion channel-targeting modulators with anticancer drugs presents a promising strategy to overcome drug resistance. This approach can be specifically categorized into two mechanisms: enhancing drug sensitivity (**Figure 4**) and reversing drug resistance (**Figure 5**). Recent studies have revealed that ion channels are also involved in the transport of essential trace elements such as iron, zinc, and copper, which play a crucial role in tumor development and drug resistance. By participating in the regulation of enzyme activity and signal transduction pathways associated with cell proliferation and apoptosis, those modulators of trace elements enhance the sensitivity of anticancer drugs or reverse their resistance (**Figure 6**).

## 4. The current drugs that target ion channels

**Figure 7:**
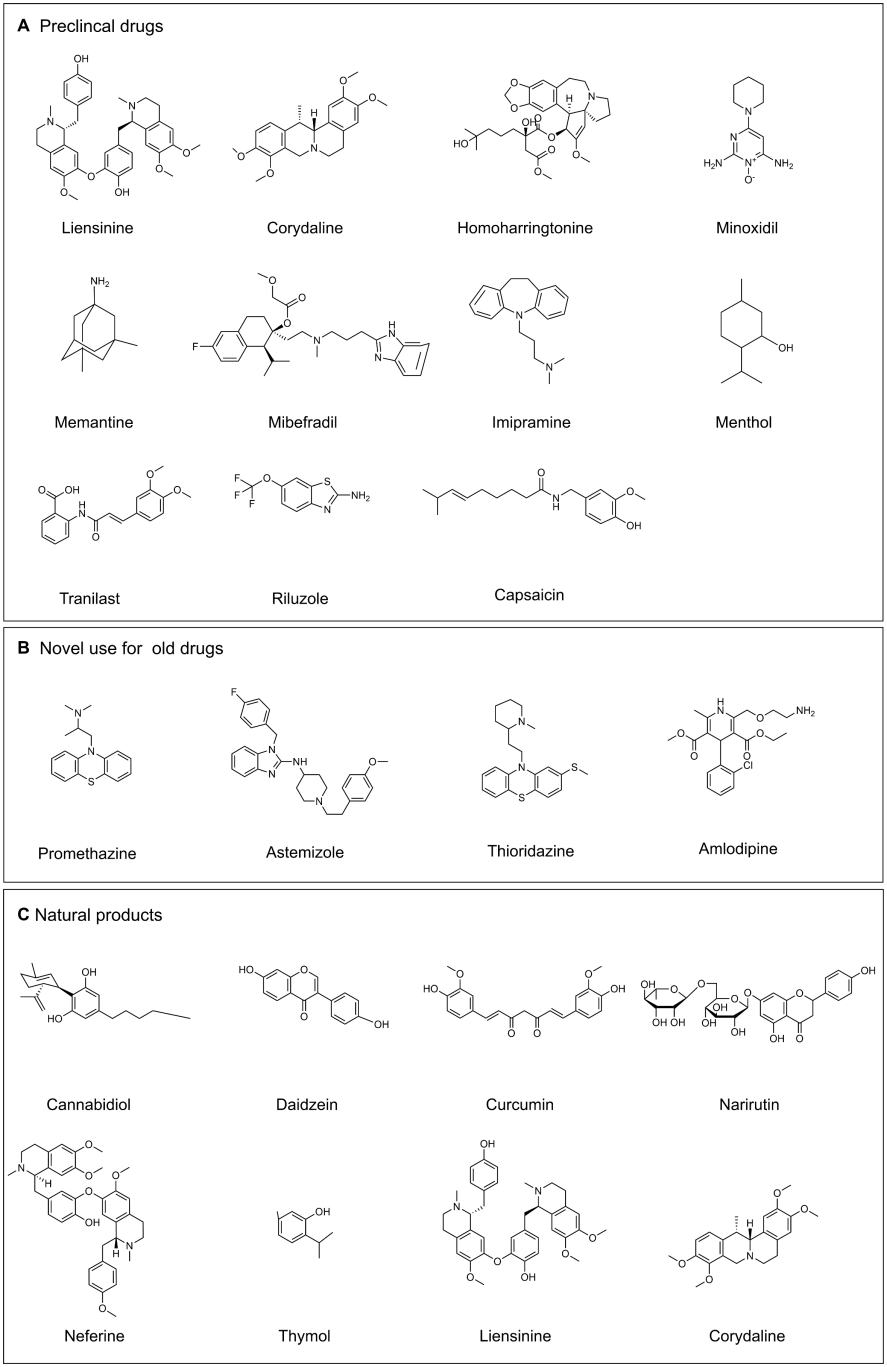
** Selected drugs that target ion channels. (A)** Preclinical drugs. **(B)** Novel use for old drugs. **(C)** Natural products. (Drawn by chemdraw)

The role of ion channels in cancer drug resistance has been increasingly highlighted in recent literature, which promotes researchers to explore the novel agents for targeting these ion channels. The ion channel modulators include preclinical drugs (**Figure 7A**), novel use for old drugs that have been approved by U.S. Food and Drug Administration (FDA) (**Table 3, Figure 7B**) and some natural products (**Table 3, Figure 7C**).

### 4.1 Novel use for old drugs that have been approved by the FDA

Ion channels are currently essential drug targets for the treatment of a variety of diseases, including type 2 diabetes, hypertension, epilepsy, arrhythmia, anxiety disorders, and cancer. The novel use of FDA-approved old drugs as Ion channel modulators is a promising approach for anticancer treatment. Ion channels are not the primary targets of imipramine or astemizole, but these drugs can block various channels due to their high affinity. Specifically, imipramine blocks sodium, potassium, and calcium ion channels, while astemizole affects several potassium channels associated with Kv10.1 189. Promethazine, known as a nonspecific Eag1 (K_V_ 10.1) blocker, can inhibit the growth of SCLC, pancreatic neuroendocrine tumors, and Merkel cell carcinoma 190. Additionally, cisplatin-resistant tumors remain sensitive to imipramine treatment. Therefore, imipramine and other related TCAs might serve as second-line treatment for patients with SCLC refractory to cisplatin/etoposide 191. The efficacy of TCAs is attributed to their non-specific and "non-targeted" mode of action, which impacts multiple molecules on the surface of cancer cells. For instance, the use of the TCA amitriptyline has been linked to significantly prolonged overall survival of patients with brain metastases from various carcinomas or GBM 192. hEag1 blockers such as astemizole and mAb56, when combined with commonly used chemotherapeutic drugs, can increase the apoptotic response of PLB-985 cells in AML 193. The antihistamine drug astemizole shows its ability to reduce breast cancer cell proliferation through selective blockade of Eag1 channels 189. Additionally, astemizole and the antipsychotic drug thioridazine, as Eag2 channel blockers, potentially reduces the growth and metastasis of intracranial xenograft medulloblastoma 194. Calcium channel blocker analogs of antihypertensive drugs including amlodipine, felodipine, manidipine, and cilnidipine, have shown significant efficacy in inhibiting filopodial formation, directional migration, and cell invasion in breast and pancreatic cancer 195. These findings underscore the potential of repurposing existing drugs to target ion channels for cancer therapy.

### 4.2 Natural products

Long-term use of chemotherapeutic drugs can produce a series of side effects, including toxicity and drug resistance 196. Natural products can serve as invaluable sources for studying biological systems and drug discovery, particularly in anticancer research 197, 198. These products have very favorable advantages due to their chemical diversity, low toxicity, safety, and availability, and can also enhance the efficacy of anticancer drugs 199. Cannabidiol (CBD), a natural product that activates the TRPV2 channel, increases the sensitivity of glioblastoma cells to cytotoxic chemotherapeutics by enhancing drug uptake. In multiple myeloma cells, CBD exhibits a synergistic effect when combined with bortezomib. Furthermore, CBD enhances the chemosensitivity in the treatment of TNBC and endometrial cancer cells 200-202. Additionally, CBD induces apoptosis in cisplatin-resistant NSCLC cells by modulating oxidative stress pathways 203. Capsaicin, another natural product and a TRPV1 agonist, triggers excessive Ca^2+^ influx leading to mitochondrial dysfunction. This process activates the mitochondrial permeability transition pore (mPTP), and initiates apoptosis in thyroid cancer cells. Capsaicin also induces autophagy in anaplastic thyroid carcinoma (ATC) cells via Ca^2+^ influx 204, 205. Thymol, a TRPV3 activator, affects Ca^2+^ homeostasis and reduces the viability of prostate cancer cells 206. Curcumin inhibits the proliferation of colon cancer cells and reduces cholesterol absorption in Caco-2 cells by activating TRPA1 channels 207. Both male and female *M. pomifera* plant extracts trigger intracellular Ca^2+^ overload via TRPV1, subsequently inducing apoptosis through multiple pathways in ER-positive MCF-7 and T47D breast cancer cells 208. Natural Borneol pretreatment enhances the sensitivity of A549 cells to low dose doxicin (DOX) and increases apoptosis. Through surface plasmonic resonance (SPR) and liquid chroorubmatography-mass spectrometry (MS-SPRI) analysis, TRPM8 is found to be a potential target for natural borneol as a sensitizer in chemotherapy 209. Neferine, a natural alkaloid, induces autophagy and reverses drug resistance by activating ryanodine receptors and promoting Ca^2+^ release 210. Narirutin, a functional food, significantly improves the therapeutic effect and eliminates the side effects of cisplatin 211. Daidzein is confirmed to inhibit the growth of lung adenocarcinoma by suppressing TMEM16A channel in a dose-dependent manner 212, 213. Homoharringtonine (HHT) is proven to be a novel TMEM16A inhibitor to suppress the growth of lung cancer 212, 213. The novel food-derived compound Liensinine can serve as a lead compound for anti-HCC drugs by targeting Kv10.1. Corydaline inhibits HCC by binding to the druggable pocket of the hEAG1 channel 214, 215. Extracts from Mallotus apelta act as novel ANO1 inhibitors to exhibit anticancer activity 216. Natural products targeting ion channels hold significant promise for cancer therapy. They are particularly effective in enhancing therapeutic outcomes and reversing drug resistance. However, their clinical application necessitates further research and validation. Ensuring safety and efficacy, as well as optimizing dosing strategies, are critical steps that must be addressed.

## 5. Conclusions and Perspectives

Although the advancements in healthcare have extended human lifespan, cancer remains a significant global health burden. Recent research highlights the intricate relationship between ion channels and cancer. Ion channels play crucial roles in cancer initiation, progression, and therapeutic response, thus termed oncogenic channels. Aberrant expression and dysregulation of ion channels have been associated with the drug resistance to conventional cancer treatment through various mechanisms. Therefore, targeting ion channels will hopefully offer a novel and promising strategy for overcoming cancer drug resistance.

Despite the identification of ion channels whose expression and/or functional alterations promote our understanding for cancer drug resistance, only a few therapeutic strategies targeting these channels have progressed to early-stage clinical trials (**Table 4, Figure 7A**). A viable ion channel therapeutic target for cancer must have low expression in normal tissue, high expression in tumor tissue, high selectivity with ligands, and minimal side effects 1. However, many drugs fail to meet these stringent criteria, underscoring the urgent need for clinical trials. Consequently, developing drugs that effectively target ion channels presents numerous challenges. Firstly, the crosstalk between ion channels and their biological function is complicated, which depends on cancer cell types, intracellular ion concentration, and other circumstances. For instance, both BKCa and calcium-activated chloride channel (CLCA) can be regulated by Ca^2+^. Sodium-calcium exchangers (NCX) function to pump Ca^2+^ out of the cell but Na^+^ in cellular uptake 217-219. Therefore, the ion channel modulators may exert different possibilities, which should be considered in the personalized medicine design for cancer patients. Secondly, ion channels are highly druggable targets, primarily due to their membrane localization. Ion channels are extensively distributed across various cells and tissues, and their activation or inhibition can significantly impact the function of multiple organ systems, potentially leading to unexpected anti-tumor side effects. For instance, TMEM16A plays a crucial role not only in tumor progression but also in regulating chloride ion transport through epithelial cells, and modulating electrical signals in smooth muscle and specific neurons 220-222. Achieving selectivity among ion channel subtypes is challenging due to their high homology. This highlights the need for cancer-specific targets and novel targeted drug delivery systems via ion channels 223. Biological agents, notably antibodies and peptides, can exhibit high selectivity for subtypes and off-targets compared to small molecules. These biological agents are metabolized normally without metabolism-mediated toxicity or drug interactions 224. The antibodies often possess strong specificity, potency, and long circulatory half-life 224-231. They can also be used for targeted cancer therapy by attaching to toxins or radioactive molecules. An antibody targeting a P2X7 variant is currently in clinical trials for basal cell carcinoma 232. The evolution of drug delivery systems highlights the need for selective cytotoxic delivery to cancer cells, which makes ion channels potential targets for anticancer drugs. Natural product-drug conjugates may provide new research avenues for prostate cancer patients over-expressing TRPV1 channel 233. Based on 3D hydrogels, limonin, a novel TMEM16A inhibitor from herbal medicine, is found to have the anticancer potential in lung cancer by targeting specific high expressed TMEM16A ion channel 234. Another natural product, Silibinin, is also found to serve as an inhibitor of TMEM16A 235. Thirdly, given the complexity of ion channel function and tumor heterogeneity, clinical decisions require precise modulation (activation or inhibition) of ion channels and personalized treatment for cancer patients. Iron metabolism has dual roles in tumorigenesis and development. Upregulation of TFRC in ADR-resistant breast cancer reverses drug resistance via ferroptosis activation, whereas increased iron absorption in ovarian cancer enhances DNA repair and drug resistance 117, 161. To address this, deepening understanding of ion channel biology, signal transduction, and their tumor microenvironment interactions is essential. Advances in electrophysiology, particularly patch-clamp technology, and mature high-throughput screening (HTS) for ion channels have accelerated ion channel research and drug discovery. At last, effective biomarkers predicting the sensitivity or resistance of cancer cells to ion channel modulators are lacking. Thus, it is essential to discover and validate these biomarkers through genomics, proteomics, and metabolomics.

In summary, addressing the challenge of cancer resistance through targeting ion channels involves several key issues. Moreover, the current lack of sufficient clinical data, which limits the practical application of these strategies. Future advancements in technologies such as high-throughput sequencing, single-cell analysis, and structural biology are expected to enhance our comprehension for the molecular mechanisms by which ion channel modulators overcome drug resistance. Additionally, progress in drug design, delivery systems, biomarker development, and personalized treatment will be essential. Although these issues, we think targeting ion channels will hopefully provide us new treatment methods to prevent or combat the emergence of drug resistance.

## Figures and Tables

**Table 1:** The ion channels involved in cancer and their molecular mechanisms.

Types of ion channels	Cancer Type	Cancer cell lines	Biological effect	Molecular mechanisms	Ref
Na^+^ (SCNN1B)	Gastric	AGS, BGC823, MKN45	Apoptosis↑, Proliferation↓	GRP78↓, PERK↑, ATF4↑, XBP1s↑, CHOP↑	11
Na^+^ (SCNN1B)	Colorectal	DLD1, SW1116	Apoptosis↑, Proliferation↓	c-Raf↓, p-ERK↓, p-AKT↓, p-MEK↓	12
Na^+^ (Nav1.5)	Oral	HSC-3	Proliferation↑	β-catenin↑, c-Myc↑, Cyclin D1↑	13
K^+^ (KCNMA1)	Gastric	MGC803, BGC823	Apoptosis↑	PTK2↓	17
K^+^ (KCNMA1)	Endometrial	Ishikawa	Proliferation↑	MEK/ERK↑	18
K^+^ (IKCa1)	Prostate	LNCaP, PC-3, DU-145	Proliferation↑	Ca^2+^↑	19
K^+^ (HERG1)	Breast	SKBR3, MDA-MB-231	Proliferation↓	p21↑, p16INK4a↑	28
K^+^ (Kv1.3)	Melanoma	B16F10	Apoptosis↓	Caveolin-Kv1.3 axis↑	29
K^+^ (KCNA5)	Ewing sarcoma, Neuroblastoma	HuVEC, HL-1, TC-71	Apoptosis↑	Caspase-3↑	33
K^+^ (Kv11.1)	Breast	MCF7, MDA-MB-231	Apoptosis↑	NRF2↑, ROS↑	34
K^+^ (KCNE2)	Gastric	SGC7901	Proliferation↓	Cyclin D1↓	35
K^+^ (Kir2.2)	Prostate	PC-3	Proliferation↑	p-RelA↑, NF-κB↑, Cyclin D1↑	36
K^+^ (KCNK3)	Lung adenocarcinoma	H1975, H1299	Proliferation↓	AMPK-TXNIP↑	42
K^+^ (Kv1.3)	Melanoma	Pmel-1, B16, Mel624	Immune↑	[K^+^]_i_↑, Akt-mTOR↑	44
K^+^ (KCNAB2)	Lung adenocarcinoma	A549, H23	Immune↑	CCL2↑, CCL3↑, CCL4↑, CCL18↑, CXCL9↑, CXCL10↑, CXCL12↑	45
Ca^2+^ (Cav1.3)	Endometrial	Ishikawa	Proliferation↑	GPER↑, Ca^2+^↑, p-ERK1/2↑, p-CREB↑	49
Ca^2+^ (CACNA1E)	NSCLC	H1299, H1975	Proliferation↑	Ca^2+^↑, EGFR↑, p-Akt↑, p-Erk↑	50
Ca^2+^ (MCU)	Colorectal	HT29	Proliferation↑	RIPK1-MCU↑, Ca^2+^↑	53
Ca^2+^ (MICU1)	Ovarian	CP20, OV90	Aerobic glycolysis↑	PDH↑	57
Ca^2+^ (STIM1)	Cervical	SiHa, CaSki	Proliferation↑	p21↓, Cdc25c↑	60
Ca^2+^ (SOCE)	Gastric	BGC-803, MKN-45	Proliferation↑	MACC1↑, p21↓, Cycline D1↑	61
Ca^2+^ (Orai3)	Pancreatic	MiaPaCa2	Proliferation↑, Apoptosis↓	SOCE↓, c-PARP↓	63
Ca^2+^ (TRPC6)	Liver	Huh-7	Proliferation↑	SOCE↑, Cycline D1↑	65
Ca^2+^ (TRPC1)	Thyroid	ML-1, FTC-133	Proliferation↑	p21↓, p27↓, Cycline D2↑, Cycline D3↑, CDK6↑	66
Ca^2+^ (ORAI1)	Lung	H1299	Immune↑	Ca^2+^↑, Calpain↑, MLPH↑, SLP2-a↑, sEV PD-L1↑	67
Cl^-^ (ClC-3)	Nasopharyngeal carcinoma	CNE-2Z	Proliferation↑	p21↓, p27↓, CDK4/6↑	69
Cl^-^ (ANO1)	Breast	HCC1954, ZR75-1	Proliferation↑	EGFR↑, CAMK↑	70
H^+^ (ASIC1a)	Glioblastoma	R54, R8	Necroptosis↑	p-RIPK1↑	73
Fe^2+^ (TFRC)	Colorectal	NCM460, CT26	Ferroptosis↑, Immune↑	Fe^2+^↑, ROS↑	79
Fe^2+^ (TRPML1)	Melanoma	A375, M214, M481, M491	Proliferation↑	p-ERK↓, p-TSC2↓, p-S6K↓, mTORC1↓	83
Fe^2+^ (FPN1)	Myeloma	ARP1, OCI-MY5	Apoptosis↑, Proliferation↓	p-STAT3↓, MCL-1↓	84
Zn^2+^ (TRPML1)	Melanoma	MeWo, M12	Necrosis↑	Zn^2+^↑	88
Cu^2+^ (CTR1)	Breast	MDA-MB-231	Tumorigenesis↑	Cu^2+^↑, PDK1↑, p-AKT↑	89

**Table 2:** Targeting ion channels enhances the sensitivity or reverses the resistance of anticancer drugs.

Synergistic	Treatment	Types of ion channels	Cancer Types	Cancer cell lines	Anticancer Drugs	Biological effect	Molecular mechanisms	Ref
Enhancing the sensitivity	Riluzole	K^+^ (KCa3.1↑, Kv11.1↓)	Colorectal	HCT116	Cisplatin	Apoptosis↑, Proliferation↓	Caspase 3↑, p-AKT↓, Cisplatin uptake↑	91
	1-EBIO	K^+^ (IK1↑)	Epidermoid	KB	Cisplatin	Apoptosis↑	Caspase-3/7↑	93
	Sparfloxacin	K^+^ (HERG K^+^↓)	Colon	HCT 116, HT-29	5-FU	Proliferation↓, Apoptosis↑	-	95
	Veratridine	Na^+^ (Nav1.5↑)	Colorectal	SW480, DLD1	5-FU	Apoptosis↑	Ca^2+^↑, p53↑	96
	CCBs	Ca^2+^	Gastric	MKN45, AGS	Doxorubicin	Proliferation↓	YY1/ERK/TGF-β↓	97
	Mibeladil	Ca^2+^ (T-Type↓)	Ovarian	A2780Cis, IGROV-1	Carboplatin	Apoptosis↑, DNA damage↑	c-PARP↑, γH2AX↑	98
	siRNA/SKF96365	Ca^2+^ (Orai1↓)	Liver	HepG2	5-FU	Autophagy↑	p-AKT↓, mTOR↓, p-p70S6K↓, LC3B-II↑, Beclin-1↑, ATG5↑, p62↓	99
	CCBs	Ca^2+^ (T-Type, L-Type↓)	Ovarian	A2780/A2780-SP	Cisplatin, Paclitaxel	Proliferation↓, CSC↓, Apoptosis↑	C-caspase3↑, ABCG2↓, ALDH↓	102
	siRNA	Ca^2+^ (Orai3↓)	NSCLC	H23, A549	Cisplatin	Apoptosis↑, CSC↓	SOCE↓, p-AKT/AKT↓, Nanog↓, SOX-2↓	103
	shRNA	Ca^2+^ (α2δ1↓)	Gastric	HGC-27	Cisplatin	Apoptosis↑, CSC↓	-	104
	1B50-1 antibody	Ca^2+^ (α2δ1↓)	SCLC	H1048	Etoposide, Cisplatin	CSC↓, Tumor growth↓	ERK↓	105
	Knockout (CRISPR-Cas9)	Ca^2+^ (TRPM2↓)	Neuroblastoma	SH-SY5Y	Doxorubicin	Proliferation↓, DNA damage↑	Ca^2+^↓, FOXM1↓, E2F1↓, Cyclin B1↓, CDK1↓, PLK1↓, CKS1↓	107
	Knockout (CRISPR-Cas9)	Ca^2+^ (TRPM2↓)	AML	U937	Doxorubicin	Proliferation↓, Autophagy↓	Ca2+↓, ROS↑, ATP↓, HIF-1/2α↓、Nrf2↓, ATF4↓, CREB↓, ULK1↓, Atg5/7↓	108
	siRNA	Ca^2+^ (TRPM8↓)	Osteosarcoma	MG-63, U2OS	Epirubicin	Apoptosis↑	p-JNK↑, p-p44/p42↓	109
	HCQ	Cl^-^ (TMEM16A)	SCCHN	UM-SCC-1, OSC19, HN30, HN31	Cisplatin	Cell viability↓	ROS↓, β-catenin↓, MiTF↓	113
	siRNA	Cl^-^ (ClC-5↓)	Myeloma	ARH77, U266, SKO-007	Bortezomib	Autophagy↓	AKT-mTOR↓, Beclin-1↓, LC3B-II↓	114
	Knockout (CRISPR-Cas9)	Cl^-^ (VRAC↓)	Head and Neck	Pica	Cisplatin	Cell viability↓, DNA damage↑	-	115
	Knockout (CRISPR-Cas9)	Cl^-^ (LRRC8↓)	Colorectal	HCT116	Cisplatin/carboplatin	Drug uptake↑, Apoptosis↑	Caspase-3↑	116
	shRNA	Fe^2+^ (TFRC↓)	Ovarian	A2780, Kuramochi	Carboplatin	DNA damage↑	Fe2+↓, FTH1↓, POLQ-RAD51↑	117
	ML-SI1	Fe^2+^ (TRPML1↓)	Breast	SUM159, MCF7	Doxorubicin	Ferroptosis↑	-	121.
	siRNA	MAFG-AS1↓	Bladder urothelial carcinoma	T24, RT4	Cisplatin	Ferroptosis↑	UCHL5-PCBP2↓, FPN1↓, Fe^2+^↑, ROS↑, MDA↑	122
	shRNA	Zn^2+^ (ZIP4↓)	Pancreatic	MIA PaCa-2; AsPC-1	Gemcitabine	Cell viability↓	ZEB1↓, ITGA3↓, ITGB1↓, α3β1↓, JNK↓, ENT1↑	124
	Tetrathiomolybdate	Cu^2+^ (CTR1↓)	Ovarian	SiHa	Cisplatin	Proliferation↓	-	127
	TRAM-34	K^+^ (KCa3.1↓)	Melanoma	A-375	Vemurafenib (BRAF-TKI)	Apoptosis↑	ROS↑, Caspase-3↑	173
	NIFE	Ca^2+^ (L-type↓)	Colorectal	HCT116, SW620	PD-1	Proliferation↓, Immune↑	Ca^2+^↓, NFAT2-STAT3↓, LASP1↑, PD-L1↓, PD-1↓	184
	HEI3090	Ca^2+^ (P2RX7↑)	NSCLC	LLC	αPD-1	Tumor growth↓, Immune↑	IL-18↑, IFN-γ↑	188
	WT-iRGD	Fe^2+^ (TRPML1↓)	Prostate, breast	PC3, T47D	PD-1	Ferroptosis↑, Tumor growth↓	TRPML1-ARL8B↓, CD4 T↑, CD8 T↑	185
Reversing the resistance	shRNA	K^+^ (Kir2.1↓)	SCLC	H69AR, H446AR	Adriamycin	Apoptosis↑, Proliferation↓	MRP1/ABCC1↓	129
	siRNA	K^+^ (KCNQ1OT1↓)	AML	HL60/ADR, K562/ADR	Adriamycin	Apoptosis↑, Proliferation↓, Migration↓, Invasion↓	miR-193a-3p↑, Tspan3↓, MRP1↓, P-gp↓, LRP↓	130
	CCBs	Ca^2+^ (L-type↓)	Pancreatic	PANC-1-GR	Gemcitabine	Apoptosis↑	ERK↓, C-caspase-3↑	131
	shRNA	Ca^2+^ (Cav3.1↓)	Glioblastoma	A172-RC1/RC2	Temozolomide	Apoptosis↑	p62/SQSTM1↓	138
	Plasmid	Ca^2+^ (Orai3↑)	Breast	T47D	Cisplatin, 5-FU, Paclitaxel	Chemoresistance↑	PI3K/Sgk-1/Sek-1↑, p53↓	139
	AMG9810	Ca^2+^ (TRPV1↓)	Cervical	CaSki CR, SiHa CR, HeLa CR	Cisplatin	Apoptosis↑, Autophagy↓,	LC3B↓, EGF↓, p-EGFR↓, p-AKT↓, MCL1↓	143
	Tranilast	Ca^2+^ (TRPV2↓)	Gastric	KATO-III	Cisplatin	Apoptosis↑	Ca^2+^↓, c-PARP↑, CASP3↓	146
	siRNA	Ca^2+^ (TRPC5↓)	Breast	MCF-7/ADM	Adriamycin	Proliferation↓, autophagy↓	Ca^2+^↓, p-CaMKKβ↓, p-AMPKα↓, p-mTOR↑, LC3-II↓	147
	ML-SI1	Ca^2+^ (TRPML1↓)	Ovarian	OVCAR8	Cisplatin	Cell viability↓	Arginine↓, Glutamic acid↓, Cysteine↓, Creatine↓	148
	siRNA	Cl^-^ (ClC-3↓)	Lung, breast	MCF-7/DOX, A549/Taxol	Taxol, DOX	Chemoresistance↓	NF-κB↓, P-gp↓	150
	siRNA	Cl^-^ (ClC-3↓)	Ovarian	A2780/PTX	Paclitaxel	Proliferation↓	MDR↓, β-tubulin↓	154
	siRNA	Cl^-^ (CLIC1↓)	Gastric	SGC-7901/VCR	Vincristine	Cell viability↓	P-gp↓, Bcl-2↓	155
	PcTx1/shRNA	Ca^2+^ (ASIC1a↓)	Liver	HepG2/R, Bel7402/R	Oxaliplatin, 5-FU	Proliferation↓, Migration↓, Invasion↓	p-AKT↓, p-GSK3β↓, Snail↓	160
	Amiloride	Ca^2+^ (ASIC1a↓)	Liver	Bel7402/FU, HepG2/ADM	5-FU, Doxorubicin	Proliferation↓	Ca^2+^↓, PI3K/AKT↓	159
	Plasmid	Fe^2+^ (TFRC↑)	Breast	MCF-7, MCF-7/ADR	Doxorubicin	Ferroptosis↑, Proliferation↓	Fe^2+^↑, MDR↑	161
	shRNA	Zn^2+^ (ZIP10↓)	Osteosarcoma	143BR	Cisplatin	Proliferation↓, Apoptosis↑	p-CREB↓, ITGA10↓, p-AKT↓, c-PARP↑, C-caspase-3↑	163
	D-penicillamine	Cu^2+^ (CTR1↓)	Ovarian	S3	Oxaliplatin	Cell viability↓	Sp1↑, hCtr1↑, p53↓, ATP7A↓, Pt/DNA↑	167
	PAPTP	K^+^ (MitoKv1.3↓)	Leukemia	B lymphocytes	Ibrutinib (BTK-TKI)	Apoptosis↑	ROS↑, Cytochrome c↑	169
	Memantine	K^+^ (Kv1.3↓)	Melanoma	MeWo, MeWo_Eto_	BSc2189 (proteasome inhibitor)	Apoptosis↑	Bak↑, Noxa↑	171
	Senicapoc	K^+^ (KCa3.1↓)	Lung	A549, A549-3R	Erlotinib (EGFR-TKI)	Proliferation↓, Migration↓	-	175
	NNC 55-0396	Ca^2+^ (TTCC↓)	Melanoma	A375, SK-MEL-28, HT144	Mibefradil (MAPK inhibitor)	Apoptosis↑, Differentiation↑	Sox2↓	178
	Mibefradil	Ca^2+^ (Cav3.1↓)	Melanoma	A375-R, M3-R	Verofenil (BRAF inhibitor)	Autophagy↓, Apoptosis↑, Migration↓	p62↑, LC3II↑	179
	D9/shRNA	Ca^2+^ (TRPM2↓)	Lung	PC-9/AR, HCC827/AR	Osimertinib (EGFR-TKI)	Apoptosis↑, DNA damage↑	Ca^2+^↓, c-PARP↑, C-caspase-3↑, ROS↑, γ-H2AX↑	180
	siRNA	Cl^-^ (ClC-3↓)	Breast	YMB-1, MDA-MB-453	anti-HER2	Drug resistance↓	HER2↓, PI3K/AKT/mTOR↑, p-STAT3↓	182

**Table 3:** Two different types of drugs target ion channels to treat cancer.

Drug name	Drug	Cancer type	Target	Mechanism	Ref
Novel use for old drugs	Imipramine, Promethazine	Lung, Pancreatic neuroendocrine tumors, Merkel cell carcinoma	hEag1 inhibitor	Induce apoptosis and reverse chemotherapy drug resistance	189, 191
	Amitriptyline	Glioblastoma multiforme	Kv10.1 inhibitor	Prolong the overall survival of patients	192
	Astemizole, Imipramine	Acute myeloid leukemia	hEag1 inhibitor	Induce apoptosis, increase chemotherapy sensitivity in PLB-985 cells	193
	Thioridazine	Medulloblastoma	EAG2 inhibitor	Reduced MB growth and metastasis	194
	Amlodipine, Felodipine, Mannipine, Cilnidipine	Breast, Pancreatic	Calcium channel inhibitor	Reduced invasion	195
Natural products	Cannabidiol	Glioblastoma, Multiple myeloma, Breast, Endometrial cancer	TRPV2 agonists	Induce apoptosis, increase chemotherapy sensitivity	200-202
	Cannabidiol	NSCLC	TRPV2 agonists	Induce apoptosis, reverse chemotherapy drug resistance	203
	Curcumin	Colorectal cancer	TRPA1 agonists	Inhibits cell proliferation and reduces cholesterol absorption	207
	Maclura pomifera	Breast cancer	TRPV1 agonists	Induce apoptosis	208
	Natural Borneol	Lung adenocarcinoma	TRPM8 agonists	Induce apoptosis, increase chemotherapy sensitivity	209
	Neferine	Colorectal cancer	RyRs agonists	Induce apoptosis, autophagy	210
	Narirutin	Lung cancer	TMEM16A inhibitor	Induce apoptosis, inhibit cell proliferation, reverse chemotherapy drug resistance	211
	Daidzein, Homoharringtonine	Lung cancer	TMEM16A inhibitor	Inhibit cell proliferation and migration	212
	Liensinine, Corydaline	Liver cancer	Kv10.1 inhibitor	Inhibit cell proliferation and migration	214, 215
	Mallotus apelta	Prostate cancer, oral squamous cell carcinoma	ANO1 inhibitor	Induce apoptosis	216
	Capsaicin	Thyroid cancer	TRPV1 agonists	Induce apoptosis, autophagy	204, 205
	Thymol	Prostate cancer	TRPV3 agonists	Decrease cell viability	206

**Table 4:** Advances in clinical trials of anticancer drugs targeting ion channels over the past few decades.

Posted	Identifiers	Interventions	Target	Cancer Types	Phase
2004	NCT00130962	ALGRX 4975	TRPV1	Neuroma	Ⅱ
2011	NCT01303341	Riluzole+Sorafenib Tosylate	VGSC	Melanoma, Advanced solid tumors	Ⅰ
2011	NCT01916317	Lidocaine+Surgery	VGSC	Breast cancer	Ⅲ
2011	NCT01298310	Lidocaine	VGSC	Morton's Neuroma	Ⅰ
2012	NCT01480050	Mibefradil Dihydrochloride+Temozolomide	TTCC	Brain and central nervous system tumors	Ⅰ
2012	NCT01578564	SOR-C13	TRPV6	Ovarian cancer	Ⅰ
2013	NCT02587819	BSCT	P2X7	Basal cell carcinoma	Ⅰ
2013	NCT01855607	Menthol	TRPM8	Breast, Gastrointestinal, Gynecological cancer	Ⅱ
2014	NCT02037464	Capsaicin	TRPV1	Prostate cancer	Ⅱ
2021	NCT05272462	Minoxidil+Platinum	Kir6/SUR	Epithelial ovarian cancer	Ⅱ
2021	NCT04801342	WBRT+Memantine	TRPM2	Brain cancer	Ⅱ
2022	NCT04863950	Imipramine Hydrochloride+Lomustine	EAG1	Glioblastoma	Ⅱ
2022	NCT05626829	Tranilast+Radiotherapy	TRPV2	Nasopharyngeal carcinoma	Ⅱ
2023	NCT06007846	Memantine	Kv1.3	Liver cancer	Ⅱ/Ⅲ
2023	NCT04761614	Riluzole+mFOLFOX6+Bevacizumab	VGSC	Colorectal cancer	Ⅰ
2024	NCT06515678	CCB+ACEi+Bevacizumab	LTCC	Ovarian cancer	-
2014	NCT02037464	Capsaicin	TRPV1	Prostate cancer	Ⅱ

## Abbreviations

AML: Acute myeloid leukemia
ADR: Adriamycin
ASIC1a: Acid sensing ion channel 1a
BKCa: The large-conductance calcium-activated potassium channel
BTB: Blood-tumor barrier
BZ: Bortezomib
BUC: Bladder urothelial carcinoma
CRC: Colorectal cancer
CTR1: Copper Transporter 1
CSC: Cancer stem cell
CCBs: Calcium channel blockers
ClC-3: Chloride channel-3
CLIC1: Chloride intracellular channel 1
CLL: Chronic lymphocytic leukemia
CBD: Cannabidiol
CLCA: Calcium-activated chloride channel
DMT1: Divalent metal transporter 1
DCs: Dendritic cells
DOX: Doxicin
ENaC: Epithelial sodium channel
EGFR: Epidermal growth factor receptor
FPN1: Ferroportin 1
FDA: Food and Drug Administration
GC: Gastric cancer
GPER: G protein-coupled estrogen receptor
GBM: Glioblastoma multiforme
GuHCl: Guanidine hydrochloride
HCC: Hepatocellular carcinoma
HCQ: Hydroxychloroquine
HHT: Homoharring-tonine
IKCa1: Intermediate Conductance Calcium-Activated Potassium Channel 1
IREB2: Iron-responsive element-binding protein 2
iASPP: Inhibitor of apoptosis-stimulating protein of p53
IC_50_: Semi-inhibitory concentration
ICD: Immunogenic cell death
KCNQ1OT1: Potassium voltage-gated channel subfamily Q member 1 overlapping transcript 1
MCU: Mitochondrial calcium uniporter
MDR: Multidrug resistance
MAFG-AS1: MAF transcription factor G antisense RNA 1
MDR: Multidrug resistance
mPTP: Mitochondrial permeability transition pore
NSCLC: non-small cell lung cancer
NCCE: Non-capacitative calcium entry
NIFE: Nifedipine
NCX: Sodium-calcium exchangers
OSCC: Oral squamous cell carcinoma
PDAC: Pancreatic ductal adenocarcinoma
PcG: Polycomb proteins
PD-L1: Programmed death-ligand 1
PD-1: Programmed death-1
ROS: Reactive oxygen species
SOCE: Store-operated calcium entry
sEVs: Small extracellular vesicles
STIM1: Stromal interaction molecule 1
SPFX: Sparfloxacin
SCLC: Small-cell lung cancer
SCCHN: Squamous cell carcinoma of the head and neck
SPR: Surface plasmonic resonance
TME: Tumor immune microenvironment
TRPC: Ttransient receptor potential canonical
TFR: Transferrin receptor
TCAs: Tricyclic antidepressants
TCR: T-cell receptor
TRPML1: MCOLN1/ mucolipin TRP channel 1
TNBC: Triple-negative breast cancer
TRPM2: Transient receptor potential melastatin-2
TMCO1: Transmembrane and coiled-coil domains 1
TKIs: Tyrosine kinase inhibitors
TTCC: T-type calcium channel
TREGs: Regulatory T cells
UPR: Unfolded protein response
VGSCs: Voltage-Gated Sodium Channels
VRACs: Volume-regulated anion channels
